# Care needs of chronically ill patients with intellectual disabilities in Dutch general practice: patients’ and providers’ perspectives

**DOI:** 10.1186/s12913-024-11155-0

**Published:** 2024-06-14

**Authors:** Milou van den Bemd, Monique Koks-Leensen, Maarten Cuypers, Geraline L. Leusink, Bianca Schalk, Erik W. M. A. Bischoff

**Affiliations:** Department of Primary and Community Care, Radboud University Medical Centre, Geert Grooteplein Zuid 10, Nijmegen, 6525 GA The Netherlands

**Keywords:** Intellectual disability, General practice, Diabetes mellitus, Cardiovascular disease, Chronic obstructive pulmonary disease, Delivery of healthcare, Chronic disease, Disease management

## Abstract

**Background:**

To reduce the impact of chronic diseases (cardiovascular disease, diabetes mellitus type 2, and chronic lung disease (asthma or chronic obstructive pulmonary disease (COPD)), it is imperative that care is of high quality and suitable to patients’ needs. Patients with intellectual disabilities (ID) differ from the average patient population in general practice because of their limitations in adaptive behaviour and intellectual functioning, and concomitant difficulties recognising and reacting to disease symptoms, proactively searching health information, and independently managing diseases effectively. Because of these differences, information on their care needs is essential for suitable chronic disease management (CDM). Inadequate recognition of the care needs of this vulnerable population may hamper the harmonisation of evidence-based and person-centred care, compounded by issues such as stigma, misconceptions, and diagnostic overshadowing. This study therefore aimed to explore the needs of patients with ID from perspectives of both patients and of healthcare providers (HCPs) in the context of CDM in general practice.

**Methods:**

This qualitative study recruited patients with ID for face-to-face individual interviews and HCPs for focus groups. With the Chronic Care Model as the underlying framework, semi-structured interviews and focus-group guides were defined to explore patients’ care needs and HCPs’ perspectives. All interviews and focus groups were audio-recorded and transcribed verbatim. Using Atlas.ti software, data were analysed using reflexive thematic analysis.

**Results:**

Between June and September 2022, 14 patients with ID and cardiovascular disease, diabetes mellitus type 2, and/or asthma/COPD were interviewed; and 32 general practitioners and practice nurses participated in seven focus groups. We identified six care needs underpinning suitable CDM: trusting relationship between patient and HCP; clear expectations about the CDM process; support in disease management; directive decision-making; support in healthy lifestyle; accessible medical information.

**Conclusions:**

This vulnerable patient population has complex care needs that must be acknowledged for suitable CDM. Although HCPs largely recognise these needs, organisational factors and lack of training or experience with patients with ID hamper HCPs’ ability to fully adjust care provision to these needs. Access to, and knowledge of, easy-language information on chronic diseases and communication guidelines could aid HCPs to facilitate patients in managing their diseases more adequately.

**Supplementary Information:**

The online version contains supplementary material available at 10.1186/s12913-024-11155-0.

## Background

To reduce the high impact of chronic diseases such as cardiovascular disease (CVD), diabetes mellitus (DM) type 2, and chronic lung disease (asthma/and or chronic obstructive pulmonary disease (COPD), it is imperative that care is of high quality and suitable to patients’ care needs [1]. To ensure ‘the most appropriate care at the most appropriate time and place in the most efficient manner’ [2], chronic disease guidelines support healthcare providers (HCPs) in delivering this care. However, principles of evidence-based medicine and person-centred medicine may yield different views on what constitutes most appropriate care (one-size-fits-all versus personalised approach, respectively) [3, 4], in particular when it concerns patient groups that differ in care needs from the average patient population.

One patient group that may require a different approach in healthcare is that of patients with intellectual disabilities (ID). Although the prevalence of ID is estimated at 1.5% in Western countries, people with ID are overrepresented in chronic disease groups, such as CVD, DM, and asthma/COPD [5, 6]. Limitations in adaptive behaviour and intellectual functioning mean that people with ID often have difficulty recognising and reacting to disease symptoms, proactively searching health information, retaining information from their HCP, and independently managing diseases effectively [7–9]. As a result, such patients often require easy-language information, support in utilising healthcare, and increased health surveillance [10–14].

These care needs have been identified mainly in contexts with aims other than chronic disease management (CDM) (e.g., palliative, hospital, outpatient, or social care [10–14]). Because of the perpetuity of CDM, and to prevent worsening of symptoms (i.e., exacerbations) and comorbidities, understanding care needs in the CDM context is essential. Inadequate recognition of the care needs of this vulnerable population may hamper the harmonisation of evidence-based and person-centred care, compounded by issues such as stigma, misconceptions, and diagnostic overshadowing, where symptoms are wrongly attributed to the ID rather than to health problems [15, 16]. Consequently, health(care) inequities between people with and without ID continue to exist. This study therefore aimed to explore the needs of patients with ID from perspectives of both patients and of HCPs in the CDM context for patients with ID.

## Methods

### Design and context

This study is qualitative, combining views of patients with ID and HCPs (general practitioners (GPs) and practice nurses (PNs)). Semi-structured individual interviews yielded an in-depth understanding of the personal experiences of chronically ill patients with ID. The focus-group setting allowed for broad exploration of HCPs’ perspectives by sharing experiences in providing care to chronically ill people with ID. Interviews and focus groups were conducted non-sequentially. The study protocol was preregistered (https://osf.io/b4er7).

In the Netherlands, general practice (GPs and PNs) plays a pivotal role in managing chronic diseases for the majority of patients with (and without) ID, offering accessible and comprehensive care [5, 17, 18]. PNs take up most tasks of chronic disease management, such as monitoring disease progression, patient education, and signalling complications. Their active involvement in chronic disease management has been shown to increase quality of care [19–21].

**Fig. 1:**
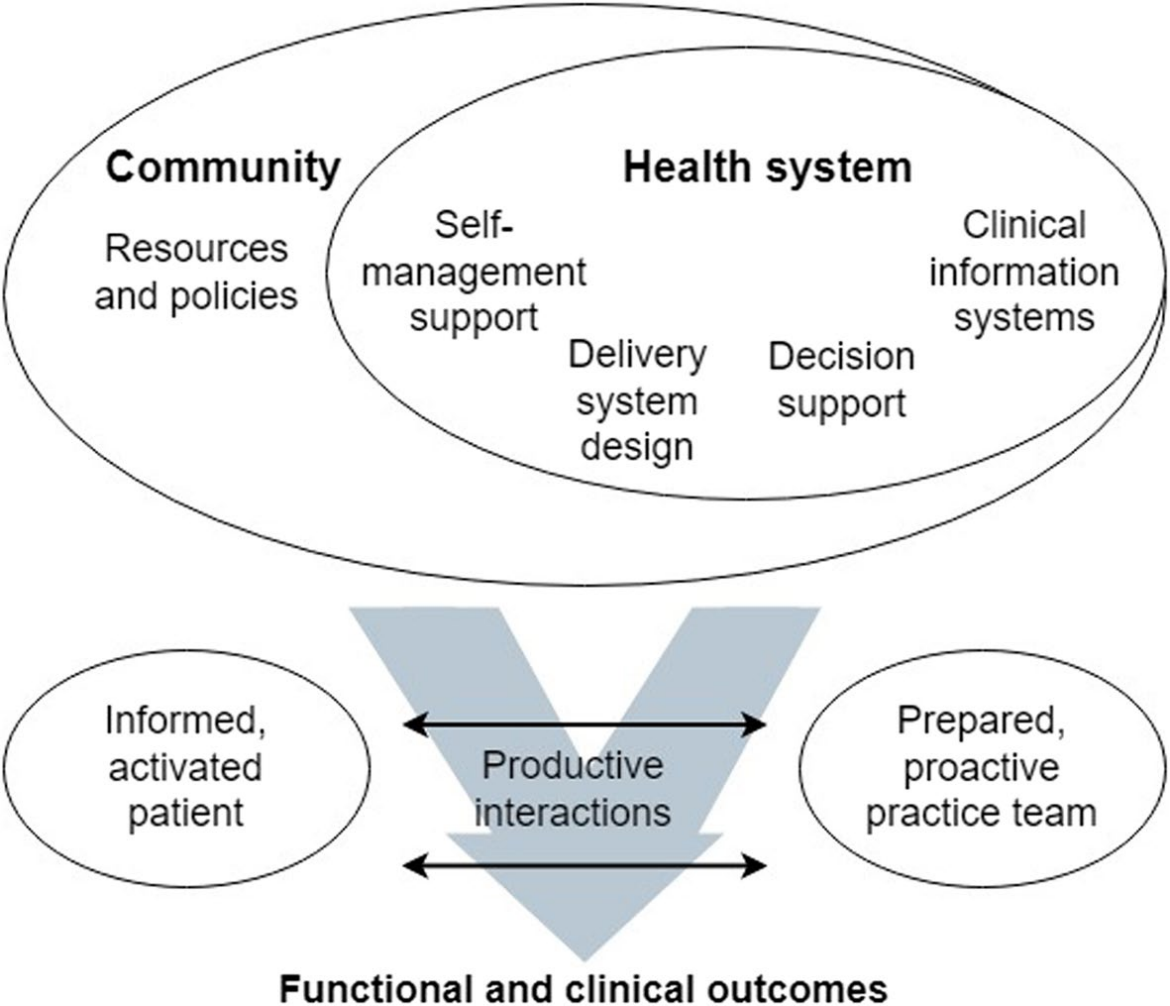
Chronic care model. The Chronic Care Model, the foundation for national care standards that specifies prerequisites for high-quality CDM [22, 23], served as the basis for the interview and focus-group guides (Fig. 1). Figure reproduced from Wagner [22]. The Chronic Care Model posits that six domains are the foundation of chronic care. In the *health system*, the structure, goals, and values should revolve around providing high-quality care to patients. Second, *self-management support* in the sense of patient education helps patients and relatives to acquire skills to manage the chronic disease adequately. Third, to aid healthcare providers (HCPs) with *decision support*, it is essential that evidence-based clinical guidelines are incorporated into practice. Fourth, the way in which *delivery systems* are designed, for instance in multidisciplinary teams, can make care more efficient. Fifth, adequate *clinical information systems* may improve compliance to guidelines or care planning. Sixth, *community resources*, such as linkages with other HCPs or community-based resources may aid in short lines of communication, through which carers and HCPs may cooperate efficiently

### Study populations and recruitment

Two distinct populations of study participants were recruited between January and September 2022.

Persons with ID were recruited who were 18 years or older, had a chronic disease (i.e., CVD, DM, and/or asthma/COPD) for which they actively received CDM, and could communicate verbally in an interview. People with borderline, mild, or moderate ID were recruited, because these groups are most likely to be able to be valuable interviewees [24]. Patients were recruited via GPs, advocacy groups for people with ID, care organisations, and snowball methods. GPs and PNs were recruited who provided CDM to patients with (suspected) ID. They were approached via flyers, email, face-to-face, education days for GPs, and snowball methods [25].

Purposive sampling was used to reflect variation in perspectives and backgrounds [26] in order to construct a holistic understanding of CDM [27]. Practically, this meant that we selected male and female patients of various ages with various chronic diseases (i.e., CVD, DM, asthma/COPD) living in various settings (i.e., in residential-care organisations, individually, or with family). In addition, we selected male and female HCPs of various ages with various experience in providing CDM to patients with ID. By closely monitoring our data collection for signs of saturation, we ensured to only approach potential participants before data saturation occurred. This way we avoided unnecessarily having to include potential participants. All study participants had to read and sign an informed consent form before participating in their interview or focus group. This study followed international guidelines for reporting qualitative research (COREQ) [28].

### Data collection

Semi-structured interview and focus-group guides (see appendix) were developed based on the Chronic Care Model and literature on care experiences of people with ID. The interview guide was developed in collaboration with a co-researcher with ID and pilot-tested in two chronically ill patients with ID. The focus-group guide was tested in a group of HCPs to ensure that no relevant themes were missed.

The first author (MvdB) conducted the interviews and moderated the focus groups, after receiving relevant training. At the beginning of each interview, the interviewer (MvdB) disclosed her position as a researcher rather than a physician, to create a safe space without any perceived (medical) hierarchy. Interviews were held face-to-face, and, if required, in the presence of a third person to help the participant answer questions or add relevant information. Focus groups were held during pre-planned education days at the university medical centre (Radboudumc) or online. The first two focus groups were observed by an assistant, who took notes and observed the atmosphere in the groups.

All interviews and focus groups (see Table 1) were audio-recorded and transcribed verbatim. When inductive thematic saturation was achieved during coding and analysis– meaning that no new codes or themes were identified– data collection was completed.

**Table 1:** Description of interview and focus-group participants

**Interviews**					
**No**	**Duration**	**Sex**	**Age**	**Living situation**	**Chronic disease**
1	37 m	Female	29	Independent living, ambulatory care	Chronic lung disease
2	66 m	Female	56	Independent living, ambulatory care	Cardiovascular disease
3^a^	48 m	Female	53	Group home	Cardiovascular disease, diabetes mellitus
4	45 m	Male	69	Independent living, no care	Cardiovascular disease, chronic lung disease
5	39 m	Female	42	Independent living, no care	Chronic lung disease
6	29 m	Male	59	Group home	Cardiovascular disease
7	41 m	Male	52	Group home	Diabetes mellitus
8	34 m	Female	19	Group home	Cardiovascular disease, chronic lung disease
9	36 m	Male	32	Independent living, ambulatory care	Cardiovascular disease
10	28 m	Male	33	Independent living, ambulatory care	Chronic lung disease
11^a^	33 m	Male	52	Independent living, ambulatory care	Cardiovascular disease
12^a^	38 m	Female	40	Independent living, ambulatory care	Diabetes mellitus
13	23 m	Male	74	Group home	Diabetes mellitus
14	31 m	Male	52	Group home	Chronic lung disease
**Focus groups**					
**No**	**Duration**	**Sex**	**Age range**	**Self-reported experience with ID**	
FG1 (GPs)	68 min	4 (2 M, 2 F)	29–46	All recently graduated; little experience with patients with ID	
FG2 (GPs)	62 min	5 (1 M, 4 F)	46–66	Most (3/5) provide care to few patients with (suspected) ID	
FG3 (GPs)	67 min	7 (3 M, 4 F)	38–55	Most (5/7) provide care to many patients with (suspected) ID	
FG4 (GPs)	53 min	4 (4 M, 1 F)	37–49	All (4/4) provide care to some patients with (suspected) ID	
FG5 (GPs)	71 min	3 (3 M, 0 F)	32–68	All (3/3) provide care to some/many patients with (suspected) ID	
FG6 (GPs)	57 min	3 (2 M, 1 F)	39–63	All (3/3) provide care to some/many patients with (suspected) ID	
FG7 (PNs)	59 min	6 (6 F)	38–60	All (6/6) provide care to many patients with (suspected) ID	

*GP* General practitioner, *PN* Practice nurse, *ID* Intellectual disabilities

^a^Third person present at interview

The research team consisted of a diverse group of academics (i.e., general practitioners, (inclusive) health scientists, sociologist, epidemiologist). It was expected that this diversity would increase quality of data analysis, as we could approach the findings from clinical point of view, while also considering the patients’ and healthcare providers’ contexts as well as the broader healthcare system.

### Data analysis

The data were analysed iteratively throughout the data collection by reflexive thematic analysis using Atlas.ti software, whereby we identified common themes in the data through consecutive coding steps [29]. This type of analysis was deemed most fitting, as it allowed for interpreting participants’ experiences, while also continuously reflecting on the researchers’ own biases, assumptions, and interpretations. It consists of several consecutive phases: 1) data familiarisation, 2) initial code generation, 3) generating (initial) themes, 4) theme review, 5) theme defining and naming, and 6) report production [30].

The first five interviews were analysed independently by MvdB, MK, and BS to iteratively shape a preliminary code tree. After five interviews, consensus was reached, and the remaining nine interviews were coded independently by MvdB and either MK or BS, further refining the code tree. Preliminary themes were then formulated. Consecutively, the focus groups were similarly coded and analysed. The first focus group was coded by MvdB, MK, and BS. The remaining focus groups were coded independently by MvdB and either MK or BS, and discussed afterwards, leading to preliminary themes resulting from the focus groups.

We first developed a code tree based on the interviews, which served as a basis for the focus group code tree and was supplemented accordingly with additional information arising from the focus groups. This resulted in numerous codes present in both populations, supplemented with information from either the interviews or focus groups. These overlapping categories from both code trees were then combined to determine themes, while acknowledging differences and similarities between the two, and while ensuring that all relevant information arisen from the data collection was considered. The preliminary themes were then combined and reshaped into overarching themes. The final six overarching themes were defined as care needs, in which we were able to include all relevant information identified from the data. These care needs were discussed with a co-researcher with ID, to include a lived experience perspective in data interpretation. After discussion and agreement by all authors, the themes were written down and supported by relevant quotes.

## Results

Between June and September 2022, 14 individual face-to-face interviews were held with patients with ID and CVD (*n* = 7), asthma/COPD (*n* = 6), and/or DM (*n* = 4). We interviewed eight males and six females, aged 19–74 years old (mean age 47.3 years; Table 1). In three interviews, a third person was present. Additionally, one focus group with PNs (*N* = 6) and six focus groups with GPs (*N* = 26; 3–7 participants per group) were conducted. The groups included 14 males and 18 females (Table 1), aged 29–68 years (mean age 47.3 years).

We identified six overarching care needs for patients (Table 2): 1) trusting relationship; 2) clear expectations about the CDM process; 3) support networks in CDM; 4) directive decision-making; 5) support in healthy lifestyle; 6) accessible medical information. We discuss these needs from the perspectives of patients, GPs, and PNs.

**Table 2:** Summary of care needs according to patients and healthcare providers (HCPs)

Theme	Explanation of theme	Patients	HCPs
Trusting relationship between HCP and patient	A trusting relationship was an essential precondition for patients’ chronic disease management	A trusting relationship was determined by HCPs’ use of language: using simple words, and addressing not just patients’ medical needs but them as a person. To enforce simple language, some patients explicitly disclosed their ID	A trusting relationship started with HCPs recognising the ID, as it indicated that they should adapt their language and approach. They felt like they should address patients’ daily life before addressing medical needs. Patients’ previous negative (care) experiences could make it more complex to build a relationship
Clear expectations about the disease management process	Patients required clarity before, during, and after disease monitoring consultations	Unclear arrangements or unmet expectations caused feelings of stress and frustration	HCPs did not always explicitly discuss expectations or assumed arrangements were clear. Although they attempted to meet expectations, to do so they required knowledge on adequate approaching and the needs of patients
Support networks in assisting with disease management	Patients’ formal and informal support networks played important roles in managing patients’ chronic diseases	Support networks were necessary control mechanisms and provided patients with reassurance. Carers often coordinated access to care and assisted with information transfer between patients and HCPs	HCPs often relied on carers for relevant information on patients’ chronic disease, and for (re-)explaining information to patients in more suitable language. It was difficult to gain an overview of patients’ support networks and each one’s roles and responsibilities in CDM
Directive decision-making processes	Patients expected HCPs to make decisions for them, but this approach was contradictory to HCPs’ preferences	The higher the HCP in the perceived medical hierarchy, the more patients valued and followed decisions and advices. Although included in decision-making processes, patients expected HCPs to make final decisions	PNs experienced difficulties because patients valued their advices less than those of GPs. Most HCPs applied a more paternalistic approach and set smaller goals to get patients to value and follow advices
Support network to assist in achieving and maintaining a healthy lifestyle	Patients and HCPs acknowledged patients’ dilemma around independence versus support needed to achieve and maintain healthy habits	Patients aspired to independence but acknowledged they required support to make healthy choices. Small lifestyle modifications were seen as great accomplishments. Their living environment could either hamper or stimulate healthy lifestyles	Bigger lifestyle modifications were necessary for patients according to HCPs, as part of adequate CDM. They attempted to mobilise support networks as much as possible to achieve healthy lifestyles
Accessible medical information	For patients and HCPs, medical information should be accessible to benefit continuity of care	It could be frustrating and confronting for patients to have to repeat their medical history to different HCPs. Online medical files were useful for checking with carers	Collaboration between care organisations was difficult with inaccessible medical information in different medical records. Determining the contents of treatment plans for chronic diseases was more complex

*ID* Intellectual disabilities, *HCP* Healthcare provider, *CDM* Chronic disease management

### 1. Trusting relationship between HCP and patient

Both patients and HCPs stated that a trusting mutual relationship was essential for patients’ CDM. Without a trusting relationship, the other identified care needs could often not be achieved.

#### Patients

For most patients, a trusting relationship was primarily determined by HCPs’ use of language:

*We have a good relationship with our GP and with the cardiologist, it’s fine simply because they speak in easily understandable language.* (P11)

The feeling of being taken seriously benefits long-term patient–HCP relationships. Patients indicated that this was mostly determined by HCPs’ listening skills, availability (in terms of time and responsiveness), and ability to reassure patients. Some patients explicitly mentioned their ID to their HCP to avoid difficult language; others suggested that they felt taken more seriously when HCPs knew about their ID diagnosis. Patients often felt safer during consultations when HCPs addressed their daily life (such as their hobby) before medical aspects. This trusting relationship facilitated information transfer for patients.

#### HCPs

Most HCPs recognised the importance of continuity of care, as it functioned as a precondition for a trusting relationship to develop. For most, a trusting relationship started with recognising the ID. In almost all focus groups, without prompts, participants first shared their difficulties experienced in timely recognition of ID, before reflecting on CDM for these patients.

A diagnosis of ID in patients’ medical records functioned as an important signal to adapt language accordingly. HCPs mentioned that they adjusted their communication by speaking calmly, using informal language, and keeping sentences short. They also used other adapted approaches: scheduling longer consultations, incorporating humour, and discussing patients’ daily life before addressing medical needs. HCPs considered patients’ negative past care experiences challenging, although these approaches helped them foster connections and create an environment for trust to develop. This allowed patients to feel at ease and share medical information more easily:

*With them [patients you’ve known longer] you know a little bit about their lives so you can comment on them, like ‘how’s your hobby?’. They all often have fun things that they do in their free time. You know that, so it’s easier to make connections or bonding. … Once you have the trust [of patients], those contacts are often much easier in terms of communication … in terms of trust that you’re there to help them.* (GP1, FG4)

### 2. Clear expectations about the CDM process

Most patients required clarity and predictability before, during, and after disease monitoring consultations (e.g., starting at the agreed time or having to take medication at home), although HCPs did not always explicitly discuss what patients could expect.

#### Patients

Patients often talked about their need for clarity. Unclear arrangements or unmet expectations could result in feelings of stress and frustration:

*[Name] had told me that the agreement was that I’d get the results of the bloodwork on Friday. … Then I was suddenly called on Wednesday. And then you start to worry. … Sorry, but then I get snappy. … Because then I don’t feel so much anger but frustration, … that you were worried for two days extra although nothing was wrong.* (P5)

Patients often expected clinical examinations (e.g., blood pressure measurements) to be performed during CDM check-ups. These examinations reassured them about their self-management skills. When a consultation unexpectedly did not include such tests, patients often failed to see the relevance of that appointment, causing feelings of frustration.

#### HCPs

Unlike patients, HCPs deemed consultations focused solely on conversation, without clinical examinations, adequate to gain information about patients’ CDM. However, HCPs monitored patients with ID more closely than other patients, by scheduling more frequent consultations and conducting clinical examinations or health checks more frequently.

*When taking someone else’s blood pressure, you say ‘send the results’. With this one you say: ‘Come back in a week to measure your blood pressure again, make an appointment.’* (GP4, FG2)

To meet patients’ expectations as well as possible (e.g., performing clinical examinations during consultations, simple-language explanation of CDM), HCPs acknowledged the need for knowledge and experience in working with patients with ID. They therefore expressed the wish for accessible training and sharing thoughts with more experienced colleagues.

### 3. Support networks in assisting with CDM

(In)formal support networks (e.g., family, care providers) had important roles in CDM at home and during consultations, even though both patients and HCPs found it difficult to identify actors involved and the responsibilities that they had in these networks.

#### Patients

Carers (individuals close to patients who support and assist them) served as the primary source for patients’ questions about their chronic disease, jointly deciding whether to consult the internet or the GP. Most patients indicated that their support networks functioned as a control mechanism and reassurance to check whether the disease was being properly managed. For instance, carers reminded patients to take medication consistently, or to order repeat prescriptions. Some patients relied on (in)formal carers to recognise symptoms of exacerbations or complications, and for medication reminders or assistance using CDM aids.

*I also have to inject [insulin] every day. … I do it myself. Because I have a device and then I can see the results. And then I tell them, and they [carers] send it to [PN].* (P13)

As patients viewed carers as accessible actors, they trusted their carers with care coordination and information transfer before, during, and/or after consultations. Patients sometimes prepared questions with their carers prior to and during GP consultations. For consultations that are deemed to be important, carers could translate information into more accessible language. The emotional and social support that carers provided to patients helped them to attend appointments.

*I once went alone [to the GP] and that went okay. But it’s nice if someone goes with me to the specialist. … A little support. I also went alone a couple of times and then I cancelled the appointment. … I didn’t feel like going alone.* (P11)

#### HCPs

HCPs indicated that they relied heavily on carers to fulfil the mediator role, supplying them with information on patients’ health complaints, (re-)explaining medical information in (more) comprehensible language, and helping to make a treatment plan. They therefore preferred carers to accompany patients during chronic disease consultations.

However, carers’ presence during consultations also posed challenges. Firstly, HCPs sometimes had to avoid engaging and making appointments exclusively with carers, who were deemed more efficient, rather than with patients themselves. Secondly, as this quote below illustrates, collaborating became more complex when carers were not medically trained, complicating information provision.

*If a carer comes along, then I hope that they’re people with some knowledge of chronic care. … I think they sometimes don’t want us to know that they have no idea what you’re talking about. So then you’re talking to two people who don’t really understand, and that’s very difficult.* (PN4, FG7)

Despite HCPs’ awareness of the networks supporting patients with CDM in daily life, HCPs’ lack of insight into actors and their responsibilities in these networks complicated care provision. GPs and PNs had different ways of handling this. PNs, who had longer consultation times than GPs, often undertook tasks that strictly did not belong in their consultation, such as assisting with taxes or with letters from their municipality. Some of the participating GPs mentioned they ideally functioned as coordinators in support networks for patients with IDs, overseeing and facilitating interactions among relevant actors within these networks. Others only preferred to fulfil a referral role, in which they directed patients to relevant healthcare professionals. As patients were limited in their capacity to self-manage their disease properly, this coordinating or referral role was deemed essential. However, as GPs often lacked time and resources for extensive support, they were sometimes left with feelings of frustration.

### 4. Directive decision-making processes

Although most patients mentioned that they expected HCPs making decisions for them, this approach was contradictory to HCPs’ preferences. Both groups were aware of (perceived) medical hierarchy and its influence on decision-making processes.

#### Patients

Patients valued HCPs’ decisions and advice based on perceived hierarchy: GPs were considered more knowledgeable than PNs, but less knowledgeable than medical specialists. However, appointments for disease monitoring with highly knowledgeable HCPs could cause more distress as patients perceived these appointments to carry highest stakes. In addition to hierarchy, comprehensible information provision and trusting relationship also affected patients’ view of HCPs’ knowledgeability.

Although patients wanted to be included in the decision-making process, most patients expected that HCPs would ultimately make the final decision for them, especially when they perceived HCPs as highly knowledgeable.

*If there are abnormalities that we have questions about, then she [cardiologist] includes me, like ‘these peaks are too high, you can do something with that’. So it’s nice that she includes me in everything that’s going to happen.* (P9)

Patients were more likely to follow advice when they perceived their HCP as knowledgeable, because they understood better the benefits of doing so.

#### HCPs

PNs seemed aware of their perceived lower status relative to GPs, as some experienced difficulties with patients valuing their decisions just as much as GPs’ decisions. Therefore, they put extra effort into building trust. Both PNs and GPs mentioned facing difficulties in exchanging information effectively during decision-making processes, including medical content and language matching patients’ cognitive abilities. PNs also mentioned that usual approaches, such as motivational interviewing, were not applicable to patients with ID. To address these difficulties, some HCPs used the teach-back method to confirm patients’ understanding. Many PNs also mentioned using visual tools – mostly pictures on how to manage diseases, such as a person injecting insulin – as their tasks more often entailed conveying practical information.

Exchanging information was deemed difficult: sometimes, HCPs realised that they had overestimated patients’ abilities, even though they were aware of patients’ ID, and sometimes HCPs assumed beforehand that they were unable to provide clear information, or patients were unable to understand information.

*I don’t have the illusion that I will do everything [right] in one go. … You incorporate those sorts of things immediately: you incorporate failure.* (GP4, FG5)

*It’s also difficult at times to judge how a person will respond. If you’re too strict, they don’t come back. And if you’re not strict enough, then they just come because it’s enjoyable.* (PN4, FG7)

Although contrary to their usual approach, most HCPs often applied a more paternalistic approach than with other patients, which they deemed necessary to provide (directive) decisions. They indicated that they provided only information that they considered most important or practical, resulting in limited shared decision-making. Others would wait until patients experienced an exacerbation, using it as leverage to encourage adherence.

Most HCPs also set smaller self-management goals for patients with ID. Although these goals did not inherently differ (e.g., maintaining a healthy lifestyle), they were often less all-encompassing, making them more achievable (e.g., taking the stairs instead of the lift). However, this approach could impact HCPs’ own motivation negatively, as progress was slower than with patients without ID.

*I actually also need small successes [with the patient] and that’s not always feasible and I find that difficult. … Yes, a good conversation can also be a small success, something like a joke or whatever, but preferably also small steps related to medicine. I find it difficult when things actually stay the same or get worse.* (GP2, FG4)

### 5. Support network to assist in achieving and maintaining a healthy lifestyle

Patients and HCPs acknowledged patients’ dilemma around independence versus support needed in achieving and maintaining healthy habits.

#### Patients

Balancing this dilemma could be difficult: despite patients’ aspiration for independence, patients acknowledged that they required sufficient internal motivation and carers’ stimulus to make healthy choices and to resist unhealthy temptations.

*Actually, I’d rather not go [dietician]. My carer says: ‘just go, just do it because it is important for you’. … In hindsight, I kind of agree with them. I do need a little push.* (P11)

Patients’ living context could either facilitate or hinder healthy decisions. Unhealthy meals or shortage of staff to aid in healthy choices limited patients’ ability to achieve and maintain a healthy lifestyle.

Most patients perceived small lifestyle modifications as great accomplishments (i.e., eating fewer unhealthy snacks), although not having access to resources could hamper healthy living. For many patients, essential resources targeted specifically at people with (intellectual) disabilities, such as lifestyle consultants, dieticians, or suitable sport clubs, were not always accessible, either financially or practically:

*[Swimming] is the only sport I can still do because of my back. … Yes, everything in life is getting more expensive. And there are funds where you can get money from your municipality to do recreational things, but still … I [also] have to pay a taxi with that money …. It’s not always easy, but I try.* (P12)

#### HCPs

Despite patients’ small lifestyle achievements, HCPs often expected or wanted bigger changes, leaving them with a dilemma on how to address lifestyle. Some mentioned that they were cautious, as patients often already faced multiple problems in different life domains simultaneously. Some HCPs dealt with this hesitation by not discussing lifestyle at all or by being more lenient with protocols for discussing lifestyle with patients with ID than for patients without ID.

*Obviously, you’re supposed to do and ask and examine a number of things [at a consultation]. And you can do the examination while you chat. … And while you’re talking, you often hear information about all of the things you have to tick off.* (PN4, FG7)

HCPs that did address lifestyle mobilised existing support networks as much as possible to attain small goals, which could aid in providing incentives and motivation to keep agreements.

### 6. Accessible medical information

Both patients and HCPs expressed their preference for accessible medical information, as it would benefit continuity of care.

#### Patients

Some patients found it frustrating to repeat their medical history, especially when there was no continuity of care:

*I’ve seen three cardiologists in two years, never the same one. … Then you have to tell the whole story again. And I say, people, you have the file, just open it. You get tired of that sometimes and then I think to myself, I’ll tell my sister that I’m going home because I can’t stand it here anymore.* (P6)

Patients with sufficient digital skills found it useful to check online medical files for arrangements and results of clinical examinations, or asked questions online rather than visiting or calling the general practice.

#### HCPs

HCPs from different care organisations were not always aware of the type and frequency of medication prescriptions and clinical examinations or whether a patient had a (suspected) ID, because medical records from different care organisations were not always linkable. This complicated care provision for them, as it was complex to determine the content of treatment plans for chronic diseases.

*We had about three different medication lists for each patient. The pharmacy had one thing, I had something else, and the ID physician had something else again. Just to show that it’s far from being integrated, which is also a point, of course, and then we make mistakes.* (GP4, FG6)

## Discussion

This qualitative study explored needs of patients with ID and their HCPs, identifying several important care needs for suitable CDM. Care needs included a trusting relationship, clear expectations, support in disease management, directive decision making processes, support in healthy lifestyle, and accessible medical information. These findings can be situated within the broader context of existing literature.

The importance of a trusting relationship between patients and HCPs is well-documented in the literature. It is mainly reported that a trusting relationship leads HCPs to be more watchful for patients’ care needs [31, 32], and facilitates patients in discussing their health problems more easily [33, 34]. For patients with a chronic disease [35, 36], including patients with ID [33, 37, 38], long-term trusting relationships have been reported to improve health outcomes, healthcare use and effective care provision. For patients with both diagnoses – a chronic disease and an ID – continuity of care is thus even more essential. As people with ID often have previous care experiences of miscommunication, unclarities regarding treatment, and feelings of not being taken seriously [39], building trust requires continuity of care and time investment [40–43].

In this study, patients inherently linked trusting relationships with suitable communication, reinforcing previous research that underlines the relevance of suitable communication for high-quality (primary) care provision [34, 37, 41, 44–48]. Although recognising ID is essential to adapt communication approaches accordingly, HCPs often lack knowledge on patients with ID and on suitable adapting communication approaches [34, 37, 41, 44–48]. This is in line with studies reporting that the diagnosis of ID was often missing in patients’ medical record, leaving HCPs without any ‘formal’ indication of adapting approaches accordingly [49, 50]. During our data collection, several GP focus groups concentrated largely on recognising ID before enabling them to reflect on CDM in this patient population. Some GP participants mentioned that sharing experiences with other GPs about patients with ID was already eye-opening, displaying their lack of knowledge and experience with patients with ID.

Despite the plethora of research on communication difficulties between HCPs and people with ID, fewer address suitable approaches for information transfer. Such approaches are important for high-quality CDM because of patients’ dilemma around autonomy versus requiring support. During the brief encounters between HCPs and patients at the general practice, it is essential that patients leave with sufficient support and tools to manage their chronic condition and healthy lifestyle in the home setting adequately. For patients, clear communication often manifests as directive decision making, although they mainly appreciate being included in the process. As more paternalistic approaches potentially deprive patients of their capacity to engage in self-direction over their chronic disease [51], the literature highlights the promise of modified approaches like supported decision-making or modified motivational interviewing. These approaches can be useful for managing and meeting patients’ expectations, and to mitigate patients’ dilemma around autonomy versus requiring support, without necessarily adopting a paternalistic approach [52, 53].

Similar to previous qualitative studies, we reported on the crucial role of support networks in the lives of people with ID [7, 33, 54, 55]. However, some issues hamper adequate support. For instance, the responsibilities are often unclear although perhaps even more important for suitable CDM [54, 55]. In one study, multiple actors mentioned to take responsibility over a small part of patients’ health, resulting in a lack of overview and overall responsibility [55]. Involved actors therefore also require additional skills to identify relevant actors, to take responsibility, and to adequately support patients before, during, and after CDM consultations [54]. As HCPs mentioned in our study, this resulted in additional workload.

For both patients and HCPs, the need for integrated medical records is often documented [56–58]. Previous studies reported that access to patients’ medical information aids in decreasing risk at medication errors, increasing quality of collaboration, and increasing patient involvement in their own care [57]. Separate medical records that do not communicate across different care organisations means that patients often have to repeatedly share medical history and disclose their ID to different HCPs. This impedes the building of a trusting relationship with HCPs, an important precondition for patients with ID in effective care provision [52].

### Strengths and limitations

By including perspectives of both patients and their main HCPs, we were able to explore patients’ care needs, HCPs’ needs in providing adequate CDM, and similarities and differences in these groups’ perspectives. Although we included a rather diverse group of patients, in terms of age, sex, living arrangement, and location of residence, most interviewees (*n* = 10/14) worked as ‘experience experts’. For this job, they had received training in communication and reflection. As this allowed them more than people without such training to voice their experiences, our findings are possibly more in-depth than otherwise. Future research is invited to explore a more broad group of chronically ill patients with ID.

As only the first two focus groups were observed by an assistant, some non-verbal cues could have been missed in the other focus groups. However, the similarity between the moderator’s and the assistant’s fieldnotes in the first two focus groups and logistical reasons (planning and timing of the focus groups) made us choose to perform the other focus groups without an assistant.

Acquaintance among GPs within the focus groups may have influenced the discussion. Although familiarity can facilitate the disclosure of sensitive topics [59], it may also induce socially desirable answers [60]. Nevertheless, an attempt was made to ensure a safe environment in at least two ways. It was explicitly stated that anonymity was guaranteed and that there were no right or wrong answers. Additionally, each group discussion included only one type of HCP (GPs or PNs). This eliminated the perceived occurring medical hierarchy within general practice, shaped by differences in responsibilities between GPs and PNs, which could possibly impact feelings of safety [61]. Consequently, the participating HCPs were very reflective and open about their (in)experiences with people with ID.

Additionally, we included a diverse group of HCPs in terms of age, sex, location, and affinity/experience with people with ID. Although the majority of GPs were located in one region of the Netherlands, the online focus groups allowed us to include a more diversely located group of HCPs. Despite recruiting PNs via different channels, the PNs who participated in the (online) group discussion all had relatively much affinity and knowledge about people with ID. Therefore, the views of the PNs included in this study might differ from those of PNs with less experience with people with ID. Having an online focus group allowed participants a safe space, with them feeling free to voice their opinions within the safety and comfort of their own homes. We also made use of features like chat, raising hands, and gallery view, to manage the discussion more efficiently and to recreate the feeling of being together in a physical space [62].

### Implications

Because we used the Chronic Care Model, the outcomes of this study can be interpreted in the light of CDM in Dutch general practice. Although this model contributes to patients being informed and activated, and HCPs being proactive and prepared [63], patients with ID require modified communication approaches and CDM goals to achieve this. With this model as the underlying framework, we found that involving carers in agreements and information transfer is even more crucial for suitable CDM in patients with ID than for those without ID. Recent pilots in the Netherlands with increased consultation times have shown promising results in terms of quality of care and satisfaction with care from the perspective of both patients and HCPs [64]. It is therefore recommended to plan increased consultation time, as this allows HCPs to develop trusting relationships with both carers and patients and to adapt communication strategies accordingly [7, 42, 65], allowing for continuity of care.

Chronic disease guidelines and CDM protocols should incorporate information on necessary modifications for suitable CDM in vulnerable patient populations, like those with ID, such as communication approaches, goal setting, and activating support networks in CDM. Incorporating ID in GP and PN training, as well as access to information or guidelines on approaching patients with ID in general practice, may enhance effective communication [37, 44, 45, 58] and thereby CDM quality. Additional training on (recognising) ID, for both HCPs and current medical students, as mentioned in several focus groups, is thus essential for effective information transfer and ID recognition [66]. Suitable communication approaches may lead towards a more person-centred approach, which can be beneficial for patients with complex care needs. As the patients in this study also mentioned, it may increase their motivation for treatment adherence and maintaining a healthy lifestyle [52, 67]. Future research can explore how HCPs can be supported in decision-making processes with these patients.

For GPs, access to, and knowledge of, easy-language information, websites, and visual tools on chronic diseases (e.g., Steffie.nl, Thuisarts.nl) is essential to facilitate patients’ understanding of the necessity of CDM. Policy should be aimed at increasing visibility of such existing tools.

Future research is encouraged to include perspectives from patients’ support networks also. Particularly views from carers who assist patients with information transfer during consultations and with CDM within the home situation, are essential for gaining a comprehensive understanding of support surrounding patients’ care needs.

## Conclusions

This study explored patients’ and HCPs’ needs in the context of CDM in general practice. Patients required a trusting relationship with their HCP, clear expectations, support in CDM and healthy lifestyle, directive decision-making, and accessible medical information. HCPs largely recognised these care needs, but organisational factors and lack of training or experience with patients with ID hampered the full adjustment of CDM to these needs. More attention in research, policy, and clinical practice is necessary to stimulate the suitability of CDM for patients with ID.

### Supplementary Information

Supplementary Material 1

## Data Availability

Restrictions apply to the availability of the interview and focus-group data. Confidentiality obligations preclude use of the data for future studies.

## Abbreviations

HCP: Healthcare provider
ID: Intellectual disabilities
CDM: Chronic disease management
CVD: Cardiovascular disease
DM: Diabetes mellitus
COPD: Chronic obstructive pulmonary disease
PN: Practice nurse
GP: General practitioner
