# Supplementary material for: Care needs of chronically ill patients with intellectual disabilities in Dutch general practice: patients’ and providers’ perspectives
Source: BMC Health Serv Res. 2024 Jun 14;24:732. doi: 10.1186/s12913-024-11155-0 (PMC11177393; doi:10.1186/s12913-024-11155-0)
Supplement: Supplementary file 1 — Supplementary Material 1 [file 12913_2024_11155_MOESM1_ESM.docx]

**Appendix: Interview and focus-group guides**

**Interviews**

Based on domains of Chronic Care Model:

Introduction:

What chronic disease(s) do you have?

Continuity of care/organisation of care:

What doctor do you have for your disease? What do you think about them?

Can you tell me what usually happens if you have to visit your doctor for a check-up for your chronic disease?

Decision support:

If you have a question about your disease, to whom do you go? Why that person?

What information did your doctor gave you when you were diagnosed?

Self-management:

What do you have to do at home for your chronic disease? Do you get help with that?

What is (not) going well?

**Focus groups**

Part 0. Introduction

- Short introduction to focus group structure
- Participants introduce themselves: name, age, profession, experience with ID
- Definition of ID and how healthcare providers (HCP) might notice.

Part 1. On ID in general:

- Short discussion of cartoon (see below) of a patient and an HCP: take a specific patient with (suspected) ID in mind, or a typical patient with ID, and fill in the speech bubbles
- Discuss and use as conversation opener on ID in general.


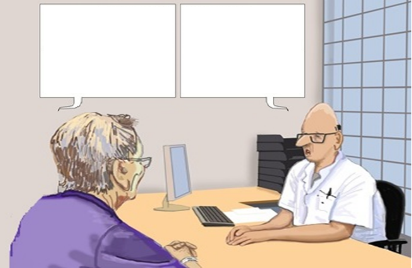


Part 2. On chronic disease management:

- To what extent are there any differences in providing chronic disease management to patients with or without an ID?
- How do you ensure that you transfer (chronic disease) information in a suitable way to a patient with ID?
- What need(s) do you have in providing chronic disease management to patients with ID?

Part 3. Care needs/wishes:

- What would you wish for your chronically ill patient with ID regarding the chronic disease management that they receive?
- What would you wish for other GPs in providing chronic disease management to patients with ID?
